# Serum DLK1 During Minipuberty and Pubertal Transition in Healthy Girls and in Girls With Precocious Puberty

**DOI:** 10.1210/clinem/dgae762

**Published:** 2024-10-28

**Authors:** Lea Vilmann, Kaspar Sørensen, Alexander S Busch, Marie L Ljubicic, Emmie N Upners, Margit B Fischer, Trine H Johannsen, Stine A Holmboe, Anders Juul, Casper P Hagen

**Affiliations:** Department of Growth and Reproduction, Copenhagen University Hospital—Rigshospitalet, Copenhagen, DK-2100, Denmark; The International Research and Research Training Centre in Endocrine Disruption of Male Reproduction and Child Health (EDMaRC), Rigshospitalet, University of Copenhagen, Copenhagen, DK-2100, Denmark; Department of Growth and Reproduction, Copenhagen University Hospital—Rigshospitalet, Copenhagen, DK-2100, Denmark; The International Research and Research Training Centre in Endocrine Disruption of Male Reproduction and Child Health (EDMaRC), Rigshospitalet, University of Copenhagen, Copenhagen, DK-2100, Denmark; Department of Growth and Reproduction, Copenhagen University Hospital—Rigshospitalet, Copenhagen, DK-2100, Denmark; The International Research and Research Training Centre in Endocrine Disruption of Male Reproduction and Child Health (EDMaRC), Rigshospitalet, University of Copenhagen, Copenhagen, DK-2100, Denmark; Department of Growth and Reproduction, Copenhagen University Hospital—Rigshospitalet, Copenhagen, DK-2100, Denmark; The International Research and Research Training Centre in Endocrine Disruption of Male Reproduction and Child Health (EDMaRC), Rigshospitalet, University of Copenhagen, Copenhagen, DK-2100, Denmark; Department of Growth and Reproduction, Copenhagen University Hospital—Rigshospitalet, Copenhagen, DK-2100, Denmark; The International Research and Research Training Centre in Endocrine Disruption of Male Reproduction and Child Health (EDMaRC), Rigshospitalet, University of Copenhagen, Copenhagen, DK-2100, Denmark; Department of Growth and Reproduction, Copenhagen University Hospital—Rigshospitalet, Copenhagen, DK-2100, Denmark; The International Research and Research Training Centre in Endocrine Disruption of Male Reproduction and Child Health (EDMaRC), Rigshospitalet, University of Copenhagen, Copenhagen, DK-2100, Denmark; Department of Growth and Reproduction, Copenhagen University Hospital—Rigshospitalet, Copenhagen, DK-2100, Denmark; The International Research and Research Training Centre in Endocrine Disruption of Male Reproduction and Child Health (EDMaRC), Rigshospitalet, University of Copenhagen, Copenhagen, DK-2100, Denmark; Department of Growth and Reproduction, Copenhagen University Hospital—Rigshospitalet, Copenhagen, DK-2100, Denmark; The International Research and Research Training Centre in Endocrine Disruption of Male Reproduction and Child Health (EDMaRC), Rigshospitalet, University of Copenhagen, Copenhagen, DK-2100, Denmark; Department of Growth and Reproduction, Copenhagen University Hospital—Rigshospitalet, Copenhagen, DK-2100, Denmark; The International Research and Research Training Centre in Endocrine Disruption of Male Reproduction and Child Health (EDMaRC), Rigshospitalet, University of Copenhagen, Copenhagen, DK-2100, Denmark; Department of Clinical Medicine, University of Copenhagen, Copenhagen, DK-2100, Denmark; Department of Growth and Reproduction, Copenhagen University Hospital—Rigshospitalet, Copenhagen, DK-2100, Denmark; The International Research and Research Training Centre in Endocrine Disruption of Male Reproduction and Child Health (EDMaRC), Rigshospitalet, University of Copenhagen, Copenhagen, DK-2100, Denmark; Department of Clinical Medicine, University of Copenhagen, Copenhagen, DK-2100, Denmark

**Keywords:** DLK1, minipuberty, puberty, CPP, body fat, birth weight

## Abstract

**Context:**

Delta-like non-canonical notch ligand 1 (DLK1) is negatively associated with bodyweight. *DLK1* pathogenic variants cause central precocious puberty (CPP) and obesity, suggesting that DLK1 links the well-established association between higher body mass index and earlier pubertal onset. However, little is known about the trajectories of circulating DKL1 in healthy girls as well as in girls with precocious puberty.

**Objective:**

To evaluate longitudinal changes in circulating DLK1 concentrations in (1) full-term, singleton healthy infant girls, (2) healthy girls during pubertal transition, and (3) girls with CPP during treatment with gonadotropin-releasing hormone agonist (GnRHa).

**Methods:**

Three longitudinal studies of (1) healthy infant girls (n = 85), (2) healthy peripubertal girls (n = 15), and (3) girls with CPP before and after GnRHa treatment (n = 15). Body fat percentage calculated using the Slaughter equation, and serum concentrations of DLK1 using enzyme-linked immunosorbent assay.

**Results:**

Serum concentration of DLK1 in healthy infant girls declined significantly through the first year of life (17.6 to 9.9 ng/mL, *P* = .020). DLK1 was inversely correlated with birth weight and BF%: r = −0.220, *P* = .044, and r = −0.503, *P* < .001, respectively. DLK1 declined from 1 year prior to pubertal onset to time of first examination after pubertal onset (10.4  to 9.2 ng/mL, *P* = .004), as well as to time at the last pubertal evaluation (10.4 to 9.8 ng/mL, *P* = .006). DLK1 levels were not affected by GnRHa treatment.

**Conclusion:**

Circulating DLK1 levels declined steeply during infancy and were less pronounced through pubertal development. Due to considerable interindividual variation, DLK1 is not useful as a diagnostic marker of pubertal onset. Importantly, DLK1 was negatively associated with birth weight and body fat percentage.

The hypothalamic–pituitary–gonadal (HPG) hormone axis is transiently active during the first months of life, a period termed minipuberty. Although the exact function of minipuberty remains largely unknown, minipuberty has been suggested to play a role in germ cell maturation (1, 2), genital development (3-5), anthropometry (6), and cognitive function (7, 8) later in life. After a quiescent period during midchildhood, release of the central inhibition of pulsatile hypothalamic gonadotropin–releasing hormone (GnRH) secretion promotes gonadotropin secretion from the pituitary and the subsequent gonadal synthesis of sex steroids initiating pubertal development (9). Exactly which factors determine the onset of this pulsatile secretion is not fully understood, but a potential mediator is DLK1 (delta-like non-canonical notch ligand 1)/Pref-1 (preadipocyte factor 1). The *DLK1* gene is imprinted (paternally expressed), located on chromosome 14q32.2, and encodes the transmembrane protein DLK1/Pref-1, which plays important roles in various biological processes, including adipogenesis, neurogenesis, and regulation of cell differentiation. DLK1 belongs to the Notch ligand family and is primarily known for its inhibition of preadipocytes into mature adipocytes (10). Circulating forms of DLK1 are predominantly found as soluble, cleaved isoforms that arise from the membrane-bound precursor protein (11, 12). The expression profile of DLK1 changes from fetal life (widely and strongly expressed) to postnatal life where expression is less pronounced but still present in various tissues (eg, preadipocytes, pituitary, adrenals, gonads, nervous system, skeletal muscles, liver, pancreas, and lung tissue) (13-15). DLK1 has garnered increasing interest in recent years since paternally inherited genetic variants of DLK1 have been identified in families with nonsyndromic central precocious puberty (CPP) with a metabolic phenotype (16). The involvement of DLK1 as a regulator of tissue growth, neurogenesis, adipogenesis, and metabolic homeostasis highlights its potential contribution to growth and development from early life to adolescence and might indicate the potential role of DLK1 as a link between the well-established association between higher body weight and earlier pubertal onset in both sexes (17-20). In this study, we aimed to evaluate the dynamics of circulating DLK1 concentrations during minipuberty and through pubertal transition in healthy girls as well as in girls with CPP during gonadotropin-releasing hormone agonist (GnRHa) treatment. Moreover, we will explore the association between circulating DLK1 levels, body fat percentage (BF%), and birth weight (BW).

## Methods and Materials

### The Copenhagen Minipuberty Study

The study is a single-center, prospective, and observational longitudinal study (2016-2019) conducted at Department of Growth and Reproduction, Copenhagen University Hospital—Rigshospitalet, Copenhagen. The study has been described in details elsewhere (21-24). A total of 233 infants (114 girls) born appropriate for gestational age (AGA) were examined at birth and up to 6 times within the first year of life. At each visit, anthropometrics was recorded, and blood sampling was attempted. In the present study, a nested cohort of 85 girls were included, all with at least 1 available blood sample analyzed for DLK1. Anthropometric examinations included in this article comprised measurements of length, weight, and skin folds.

### The Copenhagen Puberty Study (COPUS) II

The Copenhagen Puberty Study (COPUS) II is a combined cross-sectional and longitudinal study (2006-2013) of healthy Danish children from the Greater Copenhagen area. A total of 1864 children (995 girls) were examined. Among these, 209 children (108 girls) were followed longitudinally and examined every sixth months for up to 6 years. All girls were evaluated by Tanner stage (25), by palpation of the glandular breast, and measurement of height and weight, as well as blood sampling was performed. Information concerning debut of menarche (yes/no) was obtained. Age at pubertal onset was defined as the age between the last examination with Tanner stage B1 and the first examination with breast development (Tanner ≥ B2). Both unilateral and bilateral breast tissue were defined as the presence of breast tissue. Breast tissue was deemed transient if it was present at 1 examination, absent at the next, and then present again at a later examination. In total 2 girls in the present cohort exhibited transient breast tissue. The present nested cohort of 15 girls had 3 to 10 examinations prior to and through pubertal development including available blood samples for DLK1 analyses.

### Cohort of Girls With CPP

A prospective clinical study (2008-2011) based on girls diagnosed with CPP and treated with GnRH agonist (GnRHa) from the outpatient clinic at the Department of Growth and Reproduction, Copenhagen University Hospital—Rigshospitalet. A detailed description of the patients is available elsewhere (26). Pubertal development was described according to Tanner criteria (25). Girls with CPP (n = 15) were initiated on long-acting GnRHa treatment (3.75 mg of leuprolide acetate) with subcutaneous injections every 28th day. The median (range) age before treatment (baseline) was 8.9 years (7.5-9.9 years). Blood samples were drawn at baseline, at re-examination after 3 months, at 12 months, and after discontinuation of treatment after 24 months or 36 months. All examinations during treatment were performed immediately before GnRHa injections.

### Body Fat Percentage

BF% was calculated in the 2 healthy cohorts using the Slaughter equation (for sum of skinfolds less than 35 mm) (27):


$$\mathrm{BF}\text{\%}=1.33(\mathrm{TRI}+\mathrm{SCP})-0.013(\mathrm{TRI}+\mathrm{SCP}{)}^{2}-2.5$$

with SCP referring to size of subscapular skinfolds measured in millimeters, and TRI referring to size of triceps skinfolds measured in millimeters.

### Serum Sampling

DLK1 was measured in 85 infant girls; individual samples per infant girl were median 2 (range 1-6). Concentration was detectable in 205/206 available blood samples. The single sample with undetectable DLK1 was from a 12-day-old infant girl. The same girl had 4 subsequent measurements within the levels of age matched peers: 16.4 (12.9-17.8) ng/mL. We consider the undetectable sample an outlier, and we have excluded the measurement from analyses.

A total of 123 serum samples from 15 healthy peripubertal girls and 54 serum samples from 15 girls with CPP were included; individual samples per girl were 9 (3-10) and 4 (2-4), respectively.

All samples were drawn from an antecubital vein and centrifuged, and serum was pipetted and stored at –20 °C until analysis.

### DLK1 Analyses

Serum DLK1 was assessed by an immunoassay (ELISA) (Immuno-Biological Laboratories, Inc. (IBL-America), Minneapolis, USA) using an ELISA kit (IBL—America Cat# IB99504, RRID:AB_2934107). All steps were performed according to the manufacturer's instructions. Per the manufacturer's data, measurement recovers an average of 92% of DLK1 when serum samples are spiked with known concentrations of human DLK1. DLK1 levels range in human healthy control serum from 0.4 to 2.5 ng/mL. As listed by the manufacturer, the detection limit was 0.336 ng/mL and the intraassay and interassay coefficients of variation were <7 and <10%, respectively. In our hands, the interassay coefficient of variation was 10%. Internal laboratory analyses based on 4 control samples measured in 2020 and again in 2023 suggest 7% higher DLK1 concentration in measurements from 2020: 9.341 ng/mL vs 10.012 ng/mL.

### Statistical Analyses

Reference ranges for DLK1 and BF% in the infant cohort were developed using the generalized additive models for location, scale, and shape (GAMLSS) (28), based on the Box–Cox transformation, which transforms data to follow a Gaussian distribution at each age. In this model, data are summarized in 3 smooth age-dependent curves, namely LMS, where the L curve adjusts for age-dependent skewness, the M curves corresponds to the age-dependent median, and S is the age-dependent coefficient of variation curve. Calculation of individual standard deviation scores (SDSs) were based on the following equation:


$$\mathrm{SDS}\;=\;\frac{{\left(X\;/\;M\right)}^{L}-1,\;\mathrm{if}\;L\neq 0}{\left(L\times S\right)}\;$$

To evaluate individual tracking of DLK1 concentrations through minipuberty, we divided the infant girls in tertiles according to mean of individual DLK1 SDS concentrations. BW SDSs were calculated using the Marsal calculation, which was adjusted for gender and gestational age (in days) (29).

Nonparametric analyses were used due to inability to obtain normal distribution of all variables. The Wilcoxon signed rank test was used to evaluate if DLK1 decreased during infancy and through puberty. The Mann–Whitney U test was used to compare DLK1 levels in different groups of girls. Spearman’s correlation was used to assess associations between DLK1, BW, and BF%.

Statistical analyses were carried out using IBM SPSS Statistics version 28. *P* < .05 was considered to be statistically significant.

### Ethics

Each study was conducted in accordance with principles of the Declaration of Helsinki. The present studies, (1) minipuberty cohort, (2) healthy peripubertal girls, and (3) CPP cohort, were approved by the regional ethics committees, (1) H-15014876, (2) KF 01 282214 and V200.1996/90, and (3) KF 11 2006-2033, as well as by the Danish Data Protection Agency: (1) RH-2015-210, I-Suite 04146, and (2) 2010-41-5042. Moreover, 2 studies were registered by ClinicalTrials.gov: (1) NCT02784184 and (2) NCT01411527.

## Results

### Infancy

**Figure 1.**
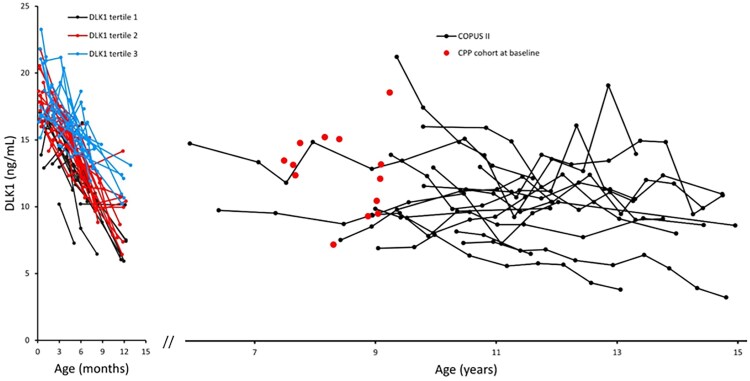
Circulating concentrations of delta-like non-canonical notch ligand 1 (DLK1) according to age in healthy infant girls (divided in tertiles according to mean DLK1 standard deviation scores), in peripubertal girls and in girls with central precocious puberty (CPP) prior to GnRHa treatment.

Median (range) serum concentration of DLK1 was 17.6 (12.9-23.3) ng/mL in healthy infant girls during the first month of life (n = 33). DLK1 declined markedly through infancy, with a median (range) concentration of 9.9 (5.9-14.2) ng/mL at 11 months of age (n = 25; *P* = .020). Each girl maintained her relative concentration of DLK1 during the first year of life (tracking) (Fig. 1) with intraindividual (within-infant) variations of DLK1 SDS (±1.4 SD, not shown) less than interindividual (between infant) variation (by definition ±2 SDS).

### From Infancy to Girls Prior to Pubertal Onset

Serum concentrations of DLK1 in late infancy did not differ from concentrations in girls prior to pubertal onset: last individual sample in the infant cohort (median age 1.0 year) vs first individual sample in the peripubertal cohort (median age 9.4 years): 10.0 (6.0-14.1) ng/mL vs 10.7 (7.3-21.1) ng/mL, *P* = 0.093.

**Figure 2.**
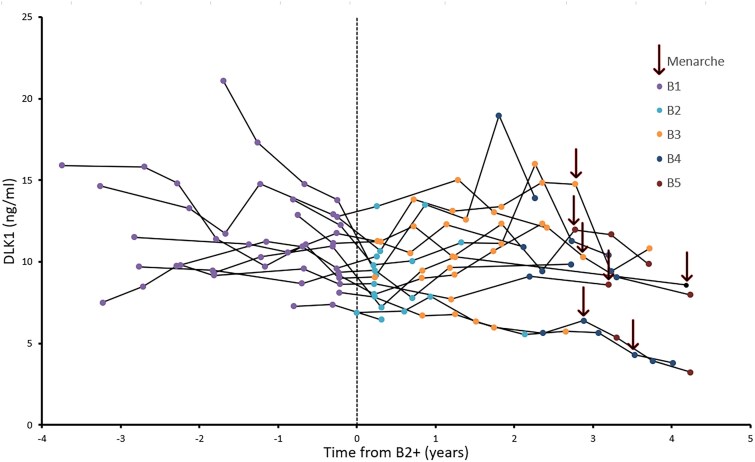
Circulating concentrations of delta-like non-canonical notch ligand 1 (DLK1) in healthy girls, according to time from puberty (+B2) and with information concerning pubertal stages (B1-B5); and black arrows indicate if menarche has occurred (yes/no).

In healthy peripubertal girls, median (range) serum concentrations of DLK1 declined from 1 year prior to pubertal onset (10.4 [7.3-14.8] ng/mL [n = 14]) to the time at first examination after pubertal onset (9.2 [6.5-13.4] ng/mL [n = 15]; *P* = .004), as well as to the last pubertal evaluation (9.8 [3.2-13.9] ng/mL [n = 15]; *P* = .006) (Fig. 2). By the last evaluation, 7 girls had reached menarche. There was no common threshold of DLK1 marking onset of puberty (Fig. 2).

**Figure 3.**
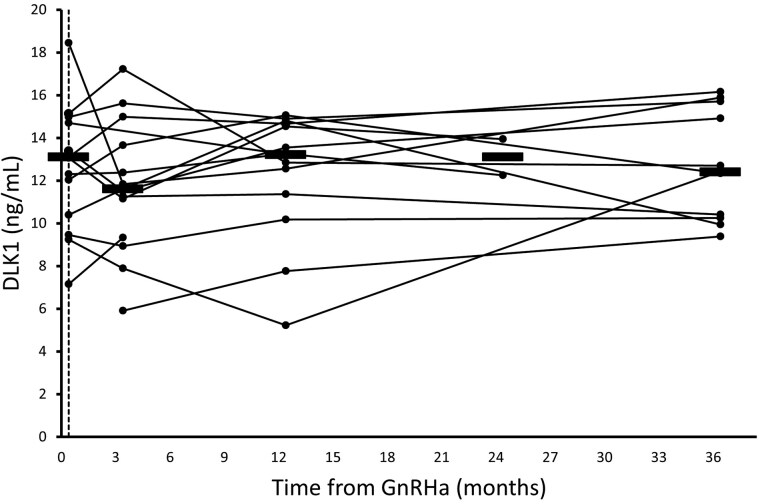
Circulating concentrations of delta-like non-canonical notch ligand 1 (DLK1) in girls with precocious puberty according to time from GnRHa treatment. Black barres represent median DLK1. Blood samples were drawn at baseline (dashed black vertical line) (n = 13), at re-examinations after 3 months (n = 14), at 12 months (n = 14), and after discontinuation of treatment after 24 months (n = 2) and 36 months (n = 11).

In girls with CPP, baseline serum concentrations of DLK1 were slightly higher than in age-matched healthy girls: 13.1 (7.1-18.4) ng/mL (n = 13) vs 9.3 (6.9-14.8) ng/mL (n = 10), *P* = .030 (Fig. 1), but the concentrations were not affected by GnRHa treatment (Fig. 3).

### Correlations Between Circulating DLK1 Levels, Body Fat Percentage, and Birth Weight

**Figure 4.**
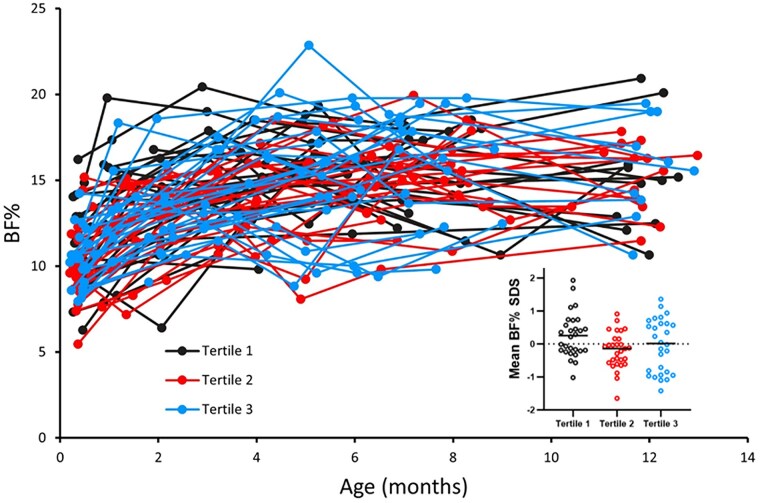
Bodyfat percentage according to age divided in mean standard deviation scores (SDSs) of delta-like non-canonical notch ligand 1 (DLK1) tertiles together with a violin plot of body fat percentage (BF%) according to mean (SDS) DLK1 tertiles.

In infant girls, the first available individual samples (age: median [25th-75th percentile] 44 [15-128] days, n = 85) of DLK1 SDS were inversely correlated with BW SDS (r = −0.220, *P* = 0.044) and BF% (r = −0.501, *P* < .001). No correlation was found with BF% at the last available serum sampling (age: 216 [152-348] days); r = −0.181, *P* = .098 (Fig. 4).

In the healthy peripubertal girls, the first available individual sample (age: median 9.4 [range 6.0-10.8] years, n = 15) of circulating DLK1 were inversely correlated with BF% (r = −0.525, *P* = 0.044). No correlation was found with BF% at the last available serum sampling (age 13.8 [11.6-15.0] years); r = −0.386, *P* = .156.

## Discussion

In this composite longitudinal cohort study, we report, for the first time, a marked decline in serum concentrations of DLK1 from infancy to pubertal onset among healthy girls. DLK1 serum concentrations further declined during pubertal transition. Due to profound interindividual variation, there was no common DLK1 threshold for initiation of pubertal onset. Circulating DLK1 concentrations were inversely associated with BW and BF%, but DLK1 was not affected by GnRHa treatment.

Postnatal concentrations of DLK1 in umbilical cord are higher than in antepartum maternal serum including breast milk, alluding to its fetal (30) or placental (31) origin. Moreover, umbilical cord DLK1 is 100-fold higher in children born small for gestational age (30) than in children born AGA, and DLK1 is significantly lower in children born large for gestational age compared with AGA (32). De Zegher et al reported that serum DLK1 had reached near-adult levels already at 4 months of age (30). These findings are in line with the DLK1 profile in the present study, revealing a steep decline during infancy followed by stable levels from late infancy to girls prior to pubertal onset and hereafter a marginal decline through puberty. The reasons for the high DLK1 concentrations after birth followed by a substantial decline are not fully understood; however, this mirrors the expression profile, which is less abundant postnatally than during fetal life. There is little knowledge of factors responsible for the termination of minipuberty; however, considering that CPP has been associated with loss of function pathogenic variants of the *DLK1* gene, we would expect high levels of DLK1 to be associated with inhibition of the HPG axis. Thus, DLK1 does not seem to be responsible for the termination of minipuberty.

DLK1 prevents the differentiation of preadipocytes into mature adipocytes and thus *Dlk1*-null mice display obesity and growth retardation (33). Conversely, mice expressing a *Dlk1* transgene in adipose tissue are lean (34). In humans, common allelic variants of the *DLK1* gene have been associated with severe obesity in children (Trio families study) (35) and circulating DLK1 is reported to be lower in obese individuals than in normal weight controls (36). Moreover, maternal serum levels of DLK1 is reduced at both 28 and 36 weeks’ gestation preceding delivery of a small for gestational age infant (31). In the present study, DLK1 was negatively associated with both BW and BF% supporting that DLK1 inhibits adipocyte differentiation.

It is well-known that obese children enter puberty earlier than lean children, and that the appetite-suppressing hormone leptin is an important link between adipose tissue and activation of puberty (19, 37). DLK1 seems to be another mediator connecting body composition and pubertal timing but, opposite to leptin, DLK1 seems to function as an inhibitor of both adipogenesis and pubertal onset (16). As previously mentioned, pathogenetic variants in the *DLK1* gene (with undetectable circulating DLK1) are seen in cases with familial CPP combined with an obese and metabolic phenotype, and the cases represented are most often girls (38-40). Moreover, genomic-wide association studies reveal that paternal inherited allelic variants of the *DLK1* gene are associated with earlier menarche (41). Although the exact incidence due to defects of *DLK1* is unknown, studies indicate that the frequency of genetic CPP due to defects of *DLK1* is not insignificant (42, 43). The exact mechanism by which DLK1 regulates pubertal timing is not fully understood, but 1 hypothesis is an indirect interfering of neurogenesis with the hypothalamic kisspeptin neuron development through the activation or inhibition of Notch target genes (16). A recent study (44) found that DLK1 levels increased from baseline and after 6 months of treatment with GnRHa in girls with CPP (from 6.5 to 7 ng/mL). In the current study, circulating DLK1 levels remained unchanged in girls with CPP during GnRHa treatment. Our results indicate that gonadotropins from the pituitary do not affect DLK1 levels. Consequently, this supports the causal relationship suggested by Mendelian randomization in genetic studies: that DLK1 regulates pubertal development, rather than DLK1 being regulated by puberty. Further studies are needed to unravel the association between circulating DLK1 and pubertal timing.

In healthy peripubertal girls, DLK1 levels declined at time of pubertal onset and as puberty progressed. This is in line with a recent study by Montenegro and colleagues (45) reporting a decline of DLK1 according to advanced Tanner stages in both boys and girls. Our results support the fact that DLK1 appears to inhibit the HPG axis centrally and thereby plays a role in the regulation of pubertal onset. However, this statement is based on our assumption that peripheral DLK1 levels reflects the levels of DLK1 at hypothalamic target tissues. Based on the results of reanalysis of a few samples after 3 years of storage at −20 °C, we cannot exclude that part of the observed pubertal decline of DLK1 could be due to evaporation and thereby concentration of DLK1 in the samples over time. In addition, the significant individual variation in DLK1 levels at pubertal onset suggests there is no universal DLK1 threshold that triggers puberty. As a result, no clinically reliable cutoff level exists to predict imminent puberty or differentiate between prepubertal and pubertal individuals. Another recent study (44) concluded that serum DLK1 levels were not a reliable biomarker for differentiating between girls with CPP and girls with premature thelarche. In general, DLK1 levels varies among different cohorts with peripubertal and pubertal individuals (36, 44, 45). These differences might be explained by inconsistency in assays used and due to the variance of developmental stage in the population groups. Further research is needed to determine whether circulating DLK1 levels could help diagnose CPP when used in larger models in which multiple factors are accounted for, such as the model by Wang et al (46) that differentiates CPP from premature thelarche.

Surprisingly, DLK1 levels in CPP girls at baseline were higher than in age-matched healthy peripubertal girls, even though the median ages in girls with CPP were at bit younger. In fact, this was contradictory to our expectations as DLK1 is considered an inhibitor of adipogenesis and premature puberty. This finding could be due to a type I error (false positive result) due to limited sample sizes and considerable interindividual variation of DLK1. Also, our CPP subjects may not represent extreme cases of CPP (very young) or familial cases. Thus, we cannot rule out that more significant findings would exist in very young girls with CPP or cases of familial CPP.

### Strengths and Limitations

This study provides detailed longitudinal evaluation of circulating DLK1 during infancy and according to pubertal development in healthy girls.

As anticipated from genetic studies on DLK1 and pubertal timing, our findings indicate that DLK1 levels decrease with the onset of puberty and continue to decline as puberty progresses. However, the analysis is based on a relatively small sample size, raising the possibility that the results could be due to chance. Despite this limitation, the results are strengthened by the thorough phenotyping of participants, which included precise information about the age of pubertal onset determined through clinical examinations by trained clinicians. Additionally, the DLK1 assay demonstrated acceptable accuracy. In the 2 peripubertal cohorts, we had relatively small sample sizes, which limited statistical power and introduced the risk of false negative results. However, we did demonstrate that DLK1 is associated with BF% as well as pubertal timing. Accurate body composition assessment is especially challenging among younger children for different reasons, including the prediction equations that tend to be specific to the population from which they were derived. However, our estimates of BF% is comparable to other populations (47). Prolonged storage at −20 °C and the precise mechanisms governing DLK1 bioavailability, half-life, and clearance in the body are not fully characterized in human physiology and should be considered when interpreting the results.

### Conclusion

Circulating DLK1 levels declined steeply during infancy and were less pronounced through pubertal development. Due to considerable interindividual variation, DLK1 is not useful as a diagnostic marker of pubertal onset. Importantly, DLK1 was negatively associated with birth weight and BF%.

## Data Availability

Data available on request from the authors.

## Abbreviations

AGA: appropriate for gestational age
BF%: body fat percentage
BW: birth weight
CPP: central precocious puberty
DLK1: delta-like non-canonical notch ligand 1
HPG: hypothalamic–pituitary–gonadal
GnRH: gonadotropin–releasing hormone
GnRHa: gonadotropin-releasing hormone agonist
